# Neutrality, conflict, and structural determinants of health in a Jerusalem emergency department

**DOI:** 10.1186/s12939-022-01681-w

**Published:** 2022-06-24

**Authors:** Zvika Orr, Levi Jackson, Evan Avraham Alpert, Mark D. Fleming

**Affiliations:** 1 Department of Nursing, Jerusalem College of Technology, 11 Beit Hadfus Street, Jerusalem, Israel; 2 Department of Emergency Medicine, Shaare Zedek Medical Center; Faculty of Medicine, The Hebrew University of Jerusalem, 12 Shmuel Bait Street, Jerusalem, Israel; 3 School of Public Health, University of California Berkeley, 2121 Berkeley Way, Berkeley, CA USA

**Keywords:** Conflict, Culture, Depoliticization, Emergency department, Healthcare professionals, Inequality, Israel/Palestine, Medical neutrality, Structural competency, Structural determinants

## Abstract

**Background:**

Medical neutrality is a normative arrangement that differentiates a zone of medical treatment disconnected from the field of politics. While medical neutrality aims to ensure impartial healthcare for all and to shield the healthcare personnel from political demands, it can also divert attention away from conflicts and their effects on health inequity. This article analyzes how healthcare professionals understand and negotiate the depoliticized space of the emergency department (ED) through their views on neutrality. It also examines how medical staff use depoliticized concepts of culture to account for differences in the health status of patients from disadvantaged groups. These questions are examined in the context of the Israeli-Palestinian conflict.

**Methods:**

Twenty-four in-depth, semi-structured interviews were conducted with healthcare personnel in a Jerusalem hospital’s ED. All but one of the participants were Jewish. The interviews were analyzed using qualitative content analysis and Grounded Theory.

**Results:**

The ED staff endorsed the perspective of medical neutrality as a nondiscriminatory approach to care. At the same time, some medical staff recognized the limits of medical neutrality in the context of the Israeli-Palestinian conflict and negotiated and challenged this concept. While participants identified unique health risks for Arab patients, they usually did not associate these risks with the effects of conflict and instead explained them in depoliticized terms of cultural and behavioral differences. Culture served as a non-controversial way of acknowledging and managing problems that have their roots in politics.

**Conclusions:**

The normative demand for neutrality works to exclude discussion of the conflict from clinical spaces. The normative exclusion of politics is a vital but under-appreciated aspect of how political conflict operates as a structural determinant of health. Healthcare personnel, especially in the ED, should be trained in structural competency. This training may challenge the neglect of issues that need to be solved at the political level and enhance health equity, social justice, and solidarity.

## Background

Medical neutrality describes an ethos in which medical personnel and their patients are positioned outside of the field of politics. Medical neutrality differentiates a zone of clinical practice and treatment of suffering in which patients are attended to impartially, and medical personnel themselves are shielded from the demands of politics and conflict [1]. However, studies of medical neutrality have deconstructed claims to a position “outside” of politics. Redfield characterizes medical neutrality as “an antipolitics with political possibilities” [1]. Denial and silencing of political conflict often divert attention away from the structural (upstream political, policy, economic, legal) causes of health inequalities and, instead, attribute inequalities to cultural and biological characteristics [2].

This article contributes to the literature on depoliticization and structural determinants of health by analyzing how medical staff in a Jerusalem Emergency Department (ED) discuss the ethos of medical neutrality. We demonstrate that medical staff endorsed the perspective of medical neutrality and understood it as a humanitarian and nondiscriminatory perspective. At the same time, some medical staff recognized the limits of medical neutrality in the context of the Israeli-Palestinian conflict. While medical staff in the ED identified unique health risks for Arab patients, most did not associate these risks with the effects of conflict and instead explained them in depoliticized terms of culture and behavior. We suggest that the normative demand for neutrality within healthcare institutions shapes how political conflict operates as a structural determinant of health.

### The Ethos of Medical Neutrality and Depoliticization

The origins of medical neutrality lie in humanitarian political and legal regimes defining the role of medical personnel and the status of the wounded in contexts of war [3]. The supposed political neutrality of medicine is undergirded by the epistemics of biomedical sciences, which represent health, disease, and suffering through universal categories [4], which inflect medical education and practice with universalist assumptions [5, 6]. This neutrality aims to make the hospital and the clinic safe havens, professional spaces where all patients get equal treatment regardless of their ethnicity, race, class, gender, religion, or other axes of difference and discrimination. In some instances, medical neutrality may even be wielded against state power, as it offers a basis for authoritative claims about the preservation of life in the face of military violence [7] or as a form of witnessing that sheds light on humanitarian violations [8]. However, the ethos of medical neutrality can also lead to overlooking the macro-level political, policy, socio-economic, and legal factors that impact patients' health [2, 9].

The ethos of medical neutrality entails depoliticization which is manifested “in processes through which objects are framed as apolitical, issues are driven outside the political realm and actors minimize, avoid or conceal the political dimension of their action” [10]. Depoliticization often prevails in contexts of conflict. Hunsmann shows how global health discourse and intervention structurally neglect conflict and minimize their political dimension even though global health work is inevitably conflict-laden [11]. In global health discourse, policymaking is usually presented as relying on neutral and objective health sciences research evidence, and politics is assumed to be distinct from policy [9, 12]. However, the concept of evidence-based policy obscures the fundamentally political nature of decision-making and the impact of politics on the creation, selection, and interpretation of evidence [9, 13].

In Israel, the supposedly depoliticized approach is dominant. Particularly, the Israeli-Palestinian conflict is not seen as a legitimate topic for in-depth discussion or action within healthcare [2]. The ethos of neutrality has been credited with enabling healthcare to become one of the most diversely integrated sectors [6]. However, Shalev explains that this neutrality, which he sees as “a shared fiction,” is applied selectively, imposing limitations on some while enabling others [14].

While Arab Israeli professionals working in healthcare recognize that the ethos of neutrality may support their inclusion within healthcare institutions, neutrality also works to mask experiences of discrimination and the unequal effects of political conflict [6]. Claims to neutrality in the medical sphere also operate as a powerful justification for ignoring unequal conditions that shape health for non-Jewish Israelis [15]. Medical neutrality is compatible with the broader practice of sanitizing institutional spaces of political discussions related to the Israeli-Palestinian conflict [16–18].

### Structural Determinants of Health and the Conflict in Jerusalem

In recent years, there has been a growing recognition of the structural determinants of health [19–21]. This concept relates to a variety of upstream, macro-level factors that produce and maintain health inequities, often in relation to race, ethnicity, religion, religiosity, class, citizenship status, language, geography, gender, and age. The structural determinants of health include public policies, laws and legal systems, social and economic systems, and political decisions and processes. However, these determinants are often misrecognized and interpreted in biologic or behavioral terms [22]. When clinicians dismiss structural factors as peripheral, they not only miss opportunities to improve health outcomes, but may also "fail at medicine’s core responsibilities to diagnose and treat illness and to do no harm" [23].

Despite the increasing awareness of the impact of structural forces on health, violent political conflict is largely overlooked in the scholarship on the topic, in medical providers' structural competency training (see below), and, more broadly, in public health, bioethics, and health promotion. Abuelaish et al. assert that although conflict always affects health, public health and health promotion experts have often failed to recognize the interrelations between health and peace [24]. This misrecognition likely stems from the dominance of the norm of neutrality, as well as from the focus on the US context in the existing literature on the structural determinants of health. The case of Jerusalem and the Israeli-Palestinian conflict contributes to bridging this knowledge gap. Specifically, our research site in a Jerusalem ED is a suitable case study as the ED is a pivotal interface between the hospital and the city's diverse communities.

Israel has a universal healthcare coverage system that includes high-quality services and technologies that are available to all residents who are recognized as "legal residents" by the state, largely free at the point of service [25]. However, irregular migrants, migrant workers, asylum seekers, and other undocumented people are not covered by the National Health Insurance Law [26, 27]. Moreover, there are health disparities between the Jewish and Arab populations in Israel [28–34]. These disparities are manifested in various health indicators, such as higher life expectancy in birth of Jews compared to Arabs (gap of 5 years among males and 3.7 years among females in 2020), and lower infant mortality rate per 1,000 live births among Jews (2.3) compared to Arabs (5.6) in 2015–2019 [32]. There are inequalities in non-communicable diseases between Jews and Arabs, across socio-economic position levels [34]. Recent studies found additional health disparities between Jewish and Arab women in Israel. For example, the prevalence of postpartum depression among Arab women is higher compared to Jewish women [33]. Palestinian-Arab perinatal women have higher anxiety and higher severity of neighborhood violence and disorder compared to Jewish perinatal women. These inequalities likely reflect residential segregation and can be reduced by changing government policies [30]. Arab Bedouin women experience multiple barriers while accessing healthcare services [29].

The Palestinian-Arab residents of East Jerusalem, who constitute around 38% of Jerusalem's population [35], have a unique status. They are entitled to Israeli health insurance, healthcare benefits, and social benefits, as long as they reside or work in Jerusalem, but there is inequity in some aspects of their healthcare [36, 37]. They have the right to vote in local elections, but they are usually unable to vote in national elections because most are permanent residents rather than citizens. Research found that they interact differently with healthcare services [38]. They are served by the city's three major general hospitals as well as smaller Palestinian hospitals in East Jerusalem. The latter suffer from a serious shortage of medicines, medical equipment, and staff due to conflict-related reasons such as the Israeli West Bank barrier (Separation barrier) that was constructed following the Al-Aqsa Intifada (Palestinian uprising) and, together with the Israeli permit regime, disconnected the East Jerusalem hospitals from numerous patients and staff from the West Bank. At the same time, the Israeli Health Maintenance Organizations (HMOs) pay these hospitals much less than they pay Israeli hospitals for health services provided to their insured patients [36, 37, 39]. These hospitals rely on international financial support and are subject to funding cutbacks due to political decisions [40].

Moreover, the poor infrastructures in East Jerusalem impact people’s health and safety. For instance, as of 2018, only 44% of the Palestinian-Arab residents of East Jerusalem had proper and legal connections to the water grid, and there is a shortage of new sewage lines in East Jerusalem [41]. According to the State Comptroller of Israel, the social and welfare services and infrastructures in East Jerusalem are inadequate, particularly those for people with disabilities, elderly people, and children at risk, and there are substantial gaps between services provided in East and West Jerusalem [42]. This affects the physical and mental health of these vulnerable populations. Additional substantial disparities exist in areas such as education, building, planning, and house demolition [41–44]. The transportation infrastructures are insufficient and often unsafe, e.g., lack sidewalks and safety barriers [41].

The Israeli-Palestinian conflict, directly and indirectly, affects the life and health of Palestinians living in East Jerusalem in multiple ways. For example, these residents face substantial delays in reaching medical care. It often takes much longer for ambulances coming from Palestinian neighborhoods to reach the ED because of conflict-related factors. Israeli medical teams often refuse to enter certain Palestinian neighborhoods in Jerusalem without a police escort due to fear of violence. In certain Palestinian areas, the residents have to use the Palestinian Red Crescent Ambulance Service which provides Basic Life Support only. In the case of a patient requiring intensive care, an Israeli ambulance staffed with a paramedic transfers the patient from the checkpoint for evacuation [45]. Additionally, Israeli army checkpoints stop ambulances on their way to the hospital from the West Bank and some of Jerusalem’s Palestinian neighborhoods [46].

The population most affected by these structural issues is the Arab residents of Jerusalem living on the Eastern side of the West Bank barrier. This population of approximately 120,000–140,000 residents constitutes 33%-39% of the city’s Arab population of 359,000 residents. Several thousand of them live in “enclaves” created along the municipal boundary of Jerusalem [41]. The Arab residents living in the neighborhoods beyond the West Bank barrier have less access to healthcare services. For example, in these neighborhoods in North-East Jerusalem, there are no Family Health Centers (Tipat Halav)^1^ [48]. The residents face difficulties reaching hospitals and sometimes even community clinics of their HMO [37]. A case in point is pregnant women in Kufr Aqab, a neighborhood located beyond the West Bank barrier. Hamayel et al. show how Israeli residency and segregation policies adversely affect the women's pregnancy and birth on physical, social, and psychological levels. Among other things, these women need to cross checkpoints to give birth in Jerusalem hospitals for their children to be eligible for permanent residency. During pregnancy, they are exposed to risky conditions and experience increased fear and anxiety. Often, their husband and family cannot accompany them to the hospital due to lack of a permit [49].

The political conflict also harms the physical and mental health of many Jewish residents of Jerusalem, such as victims of the numerous terror attacks that have taken place in the city in the past decades. For example, Ad-El et al. report that from the beginning of the Al-Aqsa Intifada in October 2000 to January 2004, 577 suicide-bombing victims were admitted to just one of Jerusalem's hospitals, suffering multiple traumas of varying severity [50]. Pat-Horenczyk screened 1,010 adolescents in Jerusalem and nearby settlements who were subjected to intensive terrorist attacks in the context of the Al-Aqsa Intifada. Two-thirds (67%) of the participants reported high levels of fear, helplessness, and horror, and 5.1% were diagnosed with PTSD. Participants also experienced functional impairment, somatic symptoms, and depression [51]. While these effects of conflict are often apparent in clinical settings, health care staff are rarely equipped with the expertise to identify and respond to conflict as a determinant of health.

### Structural Competency

Structural competency is a framework that has been developed in the past decade to help healthcare personnel identify and address structural determinants of health, such as public policies, economic systems, and healthcare delivery systems, and the ways they shape diseases, affect healthcare, and create health disparities [52]. Neff et al. define structural competency as "the capacity for health professionals to recognize and respond to health and illness as the downstream effects of broad social, political, and economic structures" [53]. Structural competency has been proposed as an additional layer of the earlier concepts of cultural competency and the social determinants of health [52].

Structural competency principles allow healthcare providers to improve treatment and patient experience, and become more engaged in policy leadership, enhancing health equity [54, 55]. Furthermore, structural competency can promote the development of critical consciousness not only in healthcare professionals but also in disadvantaged patients and community members with whom they collaborate. Critical consciousness enables stakeholders to challenge deep-seated social-political assumptions [22]. However, we suggest that political conflict is an overlooked structural determinant of health.

Metzl et al. propose that structural competency can contribute to addressing the lasting lesson of COVID-19 that "health and illness are political," hence medicine needs "to have an explicit political voice" [56]. While health care staff in many regions deal with the direct consequences of political conflict in clinical settings, understanding and addressing conflict is generally considered outside the purview of health care expertise. The dominant ethos of neutrality and the depoliticized approach exclude recognition of political conflict and may contribute to the naturalization of inequality. Naturalizing inequality refers to the "ways in which health disparities are often attributed to the behaviors or innate characteristics of the individuals or groups of people most affected by these disparities" [53]. This naturalization entails ignoring the social and political origins of health disparities [57, 58]. Depoliticization may worsen the effects of political conflict as a determinant of health.

Structural competency allows healthcare professionals to understand the process by which structurally-generated health inequalities are perceived as natural and deserved rather than unjust and imposed, and are consequently reproduced and perpetuated [19, 53]. This understanding "opens up the possibility for challenging ideologies of inequality that justify a pathogenic status quo" [19]. We propose that broadening the concept of structural competency to include structural determinants that are rooted in political conflicts can challenge the naturalization of these determinants which reproduces health inequity in conflictual settings.

## Methods

This article is based on 24 in-depth, semi-structured, individual interviews with medical staff members in the ED of a large general hospital in Jerusalem. The interviewees included 9 physicians, 9 nurses, 2 physician assistants, 3 student employees,^2^ and one employee who requested to withhold information regarding the position she held in the department. We approached all the department's physicians, nurses, physician assistants, and student employees (approximately 130 staff members) and 24 (18.5%) agreed to participate in the research. Refusal to be interviewed was usually explained by lack of time or unwillingness to discuss this study's issues. Twenty-three participants were Jewish (95.8%) and one was Arab (4.2%). This figure is similar to the share of Arabs among the physicians, nurses, physician assistants, and student employees: 5 Arab staff members who constitute 3.8% of these employees. Approximately 22% of the patients in this ED are Arab. The interviews took place in 2019 and were recorded and transcribed verbatim.

The texts were analyzed inductively using qualitative content analysis and Grounded Theory. This included an iterative search procedure for expressions and ideas [59]. The interpretative narrative analysis included several stages. Following a holistic reading of the raw data, we conducted open coding, dividing the data into many thematic categories. We subsequently identified connections between these initial categories, refined them, and combined similar categories. We chose core categories that comprised the meaningful thematic clusters around which we organized the findings [59]. Finally, after we formed the conceptual descriptions and explanations, we reread the raw data to confirm that the analysis did not depart from the data. The transcriptions were anonymized.

The qualitative interviews with the staff members are part of a broader mixed methods study in this ED that also included a quantitative survey with patients as well as qualitative interviews with patients [60, 61]. While this article focuses only on the staff, the data gathered on the patients served as useful background information.

## Results

### Medical Neutrality and Its Limits in the Israeli-Palestinian Conflict

The medical staff members referred to the theme of medical neutrality in varying ways. Most of them expressed support for neutrality as a humanitarian ethic, emphasizing that the work in the ED is non-political. However, some recognized the limitations of neutrality in everyday work and explicitly acknowledged the influence of the Israeli-Palestinian conflict on them emotionally and on the practices and discourse in the ED.

The official narrative of the hospital and the department is apolitical, neutral, striving to provide equal care to all, regardless of national identity or background. Similarly, according to the accepted narrative, efforts are made to leave the Israeli-Palestinian conflict outside the hospital perimeters, and that policy is characteristically upheld. This official narrative could be detected in the responses of several interviewees. Thus, for example, Physician 3 said the following: “From my point of view, anyone who walks through the doors of the ED is without sin. Truly, I don’t care if the person is ultra-Orthodox, Arab, a thief, or a rapist. They all get the same treatment.” Physician 8 described the interpersonal interactions following the rare occurrence of snowfall in Jerusalem as a representative instance of coexistence in the city and compared this to the hospital’s functioning: “There are generally two circumstances when Arabs and Jews tend to get along with each other: in the hospital, because we're all in the same boat, […] and when it snows and people go sledding, then everybody gets along.”

Multiple participants described the ethos of medical neutrality by invoking instances when a perpetrator and victim of a terror attack were brought to the same ED. Physician 1 said, “Even the stabber and the person stabbed can be lying in adjacent beds, separated only by the space necessary for providing treatment and they both receive the same level of treatment at the same time, without any difference, with the same degree of care.” For this and other interlocutors who described similar scenes, it seemed to be a particularly meaningful way of communicating the power of biomedical and humanitarian ethics and differentiating this orientation from the "political," which may in this example involve a military or security response (cf. [62]).

While the ED staff largely endorsed the ethos of neutrality, many participants also shared openly that they were influenced by the Israeli-Palestinian conflict. In doing so, they indicated the limits of the neutral perspective and demonstrated how the conflict impacted professional practice in the ED. For example, one staff member, who asked to withhold information regarding the position she held in the department, demonstrated an extreme attitude located at one end of the political spectrum: “First of all, I think we should not have to treat them [Arab patients] at all. In principle, this is our country. […] I am legally obligated to treat them and I won’t tell you that when I provide the care I treat them differently from the way I treat anyone else. But in my heart, I do feel that I am doing something with which I feel an internal dissonance.” This staff member repeatedly emphasized that these opinions were personal and did not represent the state of affairs in the ED.

Given the distinctions that this staff member drew between the personal and the public realms, it appears that this participant was aware of the effect of the conflict on her personal feelings even while providing treatment: “This is especially true when I encounter people whom I consider to be extremists, or in the case of prison inmates, when I know for a fact that they were involved in acts of terror […]. In my heart, I curse them; I don’t want to treat them; I know that they murdered my brethren.” This and other staff members described a negotiation with the ethos of neutrality and its limits, by creating a bifurcation between how they feel in their “heart” versus how they act.

In response to a question about obstacles to providing treatment in the hospital, the same staff member reported that, while she sometimes felt a positive connection with Arab patients, this experience was limited by political conflict: “Many times, I feel a personal connection to [Arab] patients and it’s really fun and pleasant to talk to them and for a moment I feel like this whole problem has disappeared, but I don’t want it to disappear […]. For me, it’s a matter of values.”

Participants reported that, after instances of political violence such as a terror attack, ED staff were less likely to retain a neutral perspective. Nurse 3 said, “We are no angels, but especially not on days following an attack. Those are terrible days. […] There were years when after a terror attack, no Arab patients would show up. […] In recent years, that is not the case. So you ask yourself, ‘What has changed that they no longer show this basic decency?’” It appears that this participant expected Arab patients to refrain from coming to the ED as a sign of “decency.”

### Recognizing the Differing Health Status of Arab Patients

While staff members acknowledged an ethos of medical neutrality in which they were expected to treat all patients the same, they also recognized the differing health status of Arab patients. Participants mobilized multiple forms of explanation to account for distinctive health characteristics of Arab patients, generally without reference to conflict as a structural determinant. This section will present the staff's explanations, which focus on personal behaviors, lifestyle, poverty, education, knowledge, compliance, language, and the poorer quality and lower availability of healthcare services in East Jerusalem. We then demonstrate the structural, conflict-related determinants that were largely absent from the participants' accounts.

The interviewees were asked to describe morbidity characteristics that are unique to the Arab population. Many described a wide range of chronic illnesses, such as diabetes, high blood pressure, and heart failure. Participants associated the higher rates of morbidity to a variety of factors, among the most prominent were those related to lifestyle, unhealthy behaviors, and the absence of health education. For example, Physician Assistant 1 said, “They are ill because there is no health education and it is not introduced in the early stages of life.” He mentioned an unbalanced diet and high rates of obesity. Similarly, Physician 2 explained: “The lifestyle there is catastrophic. […] There is no concern for healthy nutrition, […] no engagement in physical activity; the diet consists mostly of refined flour and sugar, […] and all the men are smokers. […] This [catastrophic lifestyle] is evident also in the number of vehicular accidents: the way they drive is much more dangerous.” In addition, participants associated Arab patients with traumatic injuries related to interpersonal violence and accidents. Some of the participants believed that there are relatively higher rates of family violence in Arab (compared to Jewish) society.

Staff members described a high frequency of visits to the ED among Arab patients and a tendency to make repeated, unnecessary visits. These practices, in the view of participants, reflect poorer quality healthcare available for Arab patients in East Jerusalem and lower levels of knowledge about how to properly use the HMO. Physician 9 explained: “Many do not receive good quality healthcare in the community because the treating physicians are not experts.” Physician 4 said: “Their HMOs are overcrowded; they have fewer expert physicians; the physicians there are less qualified, and so patients do not receive proper care there.”

According to the staff, the providers serving Arab patients were more likely to make referrals to the ED that were unjustified or unnecessary. In their view, these unnecessary referrals reflected a lower level of education and competency of both Arab patients and healthcare providers. Nurse 9 stated that she felt that “Physicians in the Arab sector referred their patients much more frequently to the hospital, rather than providing the [necessary] comprehensive care within the system.”

In addition, medical staff attributed the repeated visits of Arab patients to the poor treatment compliance of these patients. Physician 7 noted, “I have more than a few cases of Arab patients who are re-hospitalized and it turned out that they simply did not comply with the treatment as directed after the first hospitalization; thus, their condition continues to deteriorate, which is something I noticed happens more frequently in this population than in any other.” He attributed this difference to language gaps and to the fact that patients do not receive a proper explanation from the hospital’s medical staff: “Either they do not always receive an explanation or they do not always understand what they are told; it depends on the case.” Nurse 5 emphasized the gaps in knowledge and perception as the reason for poor medical compliance: “They do not quite understand how important treatment is. […] They think that the condition will simply disappear; they can take a lot; they are strong and resilient.”

In this context, some of the participants noted the fact that the language gaps prevent continuity of care between the ED and the community. Most of the ED staff members do not speak Arabic and a large proportion of the Arab patients do not speak Hebrew. Furthermore, staff members noted that the discharge papers are written in Hebrew, and not only are many of the patients unable to read Hebrew but also many of the medical staff in East Jerusalem are not sufficiently proficient in Hebrew. This has a deleterious effect on the continuity of care and leads to repeated visits to the ED.

Despite the staff’s awareness of the differences in the accessibility and quality of healthcare services in East and West Jerusalem, staff members usually did not mention the structural determinants that are behind these gaps, especially not conflict-related determinants. One of the structural determinants, for example, is the fact that in 2017, the Ministry of Health made it permissible for HMOs in East Jerusalem to outsource their services. This is forbidden anywhere else in Israel, except for East Jerusalem [63]. This exclusion arose from conflict-related tension and friction that limited the ability of Israeli HMOs to work in certain Palestinian neighborhoods in East Jerusalem. This exclusion most likely is detrimental to the quality of healthcare in East Jerusalem and increases the number of visits to the ED in the Western part of the city. This policy has often been criticized by Arab patients; however, the staff did not mention this structural factor when discussing its consequences on the ground.

Likewise, staff members recognized that Arab patients are more likely to experience poverty, and that poverty is detrimental to health, but did not make the connection to structural determinants. Physician Assistant 1 stated that “Poverty levels are high among the majority of the Palestinian population in Jerusalem and, as a result, morbidity rates are higher. At least, my impression is that the Arab patients I meet are more severely ill.” Physician 2 similarly referred to the link between health problems and socioeconomic conditions, “As [the latter] necessarily entails poorer healthcare services.” Poverty is a social determinant of health, shaped by more fundamental structural determinants such as conflict and institutional discrimination. The severe poverty in East Jerusalem^3^ derives from various structural determinants, including the West Bank barrier that separates many Palestinians in East Jerusalem from Israeli or West Bank economies, checkpoints, and other restrictions on Palestinians' mobility, planning and housing policies, the lack of citizenship status, limited access to social and welfare services, and discrimination in the labor market [41, 64, 65]. The interviewees did not discuss these structural determinants.

The poverty, as well as the knowledge and language gaps, the lack of health education, and the poor treatment compliance described by the staff, are associated with a lower level of education in East Jerusalem that is caused by policy and conflict-related factors. These factors include systematically inferior resource allocation and discrimination in investment in infrastructures in East Jerusalem (resulting, for example, in a considerable shortage of schools and classrooms in East Jerusalem), as well as the decision of most Palestinian schools not to adopt the Israeli curriculum in order not to legitimize what they and others consider the Israeli occupation of East Jerusalem, a decision that affects the ability of Palestinian youth to acquire higher education in Israel [66, 67]. However, the findings indicate that the ED staff did not go beyond the social determinants of health such as poverty – of which they were well cognizant – and did not refer to any structural-political determinants that lead to poverty, knowledge and language gaps, lack of health education, poor treatment compliance, and other phenomena that concerned them.

Interestingly, the staff did not address the issue of Arab patients' delayed arrival to the ED due to checkpoints and Israeli ambulances avoiding entering certain Palestinian areas in East Jerusalem, despite the direct relevance of this issue to ED practice. Overall, these findings point to a lack of recognition of conflict as a determinant of health and to a prevailing disregard of the entanglement of political and individual-level factors [68].

### Culture as Explanation

Participants emphasized cultural explanations to account for persistent health inequalities and conflicts with Arab patients, generally without reference to structural determinants. Medical staff in the ED demonstrated an awareness of stigmatizing representations of Arabs. Interviewees were asked directly if there exists any type of “stigma,” i.e., whether they or other staff members view the Arab population in a negative and generalizing way. While participants were aware of stigmas associated with Arab patients, it was sometimes difficult for the researchers to discern whether participants endorsed the stigmatizing perspective or distanced themselves from it. When the interviewees were asked about stigmas concerning ultra-Orthodox Jews, most explicitly stated that they did not believe the stereotype was a true reflection of reality. By contrast, when speaking about the Arab population, many of the interviewees seemingly endorsed the stigmatizing perception.

Many medical staff explicitly reproduced cultural stereotypes of Arab patients as alternatively highly respectful, on the one hand, or dramatic and violent on the other. During the interviews, physicians and nurses repeatedly referred to the theme of the respectful attitude that Arab patients and their families and communities demonstrate toward the medical staff; however, they also described them as loud, with a tendency towards violent, ill-tempered, and overly dramatic behaviors, especially as they often gather in large groups. An expression that showed up repeatedly when describing Arab patients and their tendency to interact in large groups was the term “clan” ("*Hamula*"). This is a term from the field of sociology, which in Arabic means the extended family or a small tribe that has unique social characteristics; however, Israeli slang tends to extend this meaning to convey social stigmas, such as a large, traditional, simple, and sometimes loud and vulgar family.

Some of the interviewees emphasized the overly dramatic behavior of Arab patients. Nurse 1 said the following: “There are certain stigmas, [for example] that they put on an act, that they are more dramatic,” and Nurse 2 said, “…That their reactions are extreme, beyond what the situation calls for. From my experience, I have learned to disregard the stigma and to examine each situation separately.”

Some of the interviewees tried to explain what they perceived as aggressiveness, expressed by Arab patients, as well as the respect that they show the medical staff. These two seemingly contradictory behaviors were interpreted to be a result of patients feeling strange and alienated. Student 2 explained, “It’s just that many times, the Arab patients do not feel comfortable; they do not feel free and that causes them to behave with extreme respect towards the staff.” Physician Assistant 1 attempted to analyze the factors that create the negative feelings that Arab patients experience during treatment and which, according to him, can lead to behaviors that include violence: “If people who already feel inferior come to receive treatment in a place that is foreign to them, […] then it doesn’t matter how dedicated you are or how compassionately you treat them. They come expecting to see that someone else is being treated better than them: ‘Why is he in a bed inside the room while I am sitting out here [in triage]?’ I think that is one of the reasons patients become violent. They feel that they have no other way to attract attention. Which brings us back to the Middle Eastern mentality.” The ED personnel frequently used the cultural and emotional discourses to explicate Arab patients’ behaviors and the staff’s interactions with these patients.

#### The case of “hysteria”

Members of the ED medical staff talked about specific characteristics of Arab patients who are referred to the ED. In a pilot study that we conducted, they spoke a great deal about “HY,” an expression that refers to hyperventilation but also to hysteria. Therefore, during the interviews, we asked directly about patients referred to the ED because of HY. Hyperventilation is a medical diagnosis of acute physical symptoms such as shortness of breath, chest pains, and loss of consciousness, which tend to occur in conditions of extreme mental stress, which is often referred to in the ED as hysteria. The symptoms pass when the stress subsides.

All the staff members who attempted to offer explanations for the HY phenomenon related it to cultural behavioral norms. Physician 7 said, “it is probably related to something cultural, which I cannot grasp.” Nurse 9 referred to the cultural explanation more decisively: “it is cultural.” Then she tried to analyze the concrete reason behind it: “Perhaps they are looking for some sort of attention.” The nurse then alluded to a principle that guides her therapeutic worldview: “You need to understand the underlying story.”

Staff associated HY with Arab women, although they reported that some men also presented with HY. According to Physician 7, “Arab women come into the ED as if they have fainted and are about to die.” Nurse 9 noted that HY is more frequent among young Arab women. Student 1 claimed that HY occurs *only* among Arab women, explaining this phenomenon through the following narrative: “All of the women who want attention from their husbands […] present with hysteria […] and afterward, or even during the hysteria, when the husband leaves the room, you ask her what happened and she tells you ‘Everything is okay; I had a major argument with him today’ or something of that nature.” This student, not unlike Nurse 9, related this phenomenon to the Arab culture and the relationship within the couple, which is part of the accepted cultural norms: “That is the role of the woman in the Arab population. It’s a kind of a game within the family; when a woman wants to defend herself, that is how she does it, by describing herself as suffering from some type of problem. […] They all play along. They pretend to take the treatment seriously, but actually, they all know that this is how things are done.”

Nurse 9 also pointed out the risk of adhering to stereotypes during medical treatment: “There were cases that were diagnosed as hysteria and turned out to be something completely different, such as hypoglycemia or things like that.” Physician 6 reported that, among the ED staff, there is a stereotype of “Arab women who complain of nonorganic symptoms.” However, he too stated that this stereotype may lead to misdiagnosis that would risk the patient: “Sometimes it is possible to miss serious diagnoses.”

The research participants' reluctance to discuss structural-political issues goes hand in hand with their emphasis on cultural explanations, including in the case of HY. The cultural and behavioral discourse is considered to align with the expected neutrality of the staff. Culture is perceived as unrelated to the conflict and therefore healthcare professionals feel comfortable using it. HY could be attributed to alternative underlying causes in the political and social sphere. For instance, Palestinian physicians in the West Bank depicted political and social determinants as chronic stressors that negatively affect the individual’s behavior [68]. However, in Israel these causes are more controversial and less safe than the cultural causes. The language of culture is therefore preferred and prevails among most of the ED staff.

## Discussion

This study makes two main empirical contributions to the existing literature. First, it illuminates how healthcare professionals understand and negotiate the depoliticized space of the clinic through their views on "neutrality." This includes how they view and address the limits of neutrality and how the boundaries of universal professional medical ethics are negotiated and even challenged. We suggest that the normative demand for neutrality is an under-appreciated yet important aspect of how political conflict operates as a structural determinant of health. Second, this study identifies the specific ways in which medical staff use depoliticized concepts of culture and, to a lesser extent, socio-economic development to account for differences in the health status of patients from disadvantaged groups, in this case, Arab patients. Culture serves as a safe, non-controversial (non-political) way of acknowledging problems that have their roots in politics, and of managing those problems in the context of care. The context of this study, marked by a particularly fraught "politics," offers unique insights into the question of why and how biomedicine allows "culture" into the clinic but not "politics." These insights are relevant to many other socio-political contexts worldwide.

The ethos of medical neutrality, as well as the professional field of emergency medicine, have their origins in treating the war-wounded. In this way, ED healthcare practices and the associated ethos of neutrality were both formed on the battlefield and subsequently institutionalized within hospitals. With this institutionalization, the ED medical staff confront the effects of conflict in their everyday work while also ostensibly being positioned outside of it. However, this "fiction of standing outside battle (*hors de combat*)" [3] may strengthen the naturalization of inequality as it attributes health disparities and inequity to the behaviors or innate characteristics of individuals or groups [53, 57, 58]. That is despite studies that demonstrate how in the Israeli-Palestinian context, individuals' behavioral risk factors are affected by clearly political factors [68, 69].

Although staff recognized social-economic determinants, such as poverty, education, and the poorer quality and lower availability of health services in East Jerusalem, they put stronger emphasis on culture and lifestyle. Connecting between poor health and "problematic" cultural or behavioral traits implies a tendency to "blame the victim." In this case, "blaming the victim" also clears Israeli Jews and Israeli government from responsibility for the poor health status of Palestinians in East Jerusalem.

Explanations of difference that use concepts of culture or behavior may work to manage ruptures in Arab–Jewish relations and to maintain the “political hygiene” [70] of the ED. However, we assert that in a city like Jerusalem where politics has an immense effect on patients’ lives, healthcare professionals should be cognizant of the socio-political structures that so many patients are unable to escape. For these patients, depoliticization is simply not an option. We propose that the structural competency framework, which is rather new and underused in Israel, can be effective in challenging the “depoliticization as artificial deconflictualization” [11]. Healthcare personnel, especially in the ED, should be trained in structural competency to better understand the fundamental influences on patients' health [71]. This training will allow them to move beyond the understanding of social determinants of health, such as poverty that was pointed out by many research participants, to comprehending the underlying structures that create and perpetuate the poverty of the Arab patients. Similarly, the staff’s structural competency will potentially encourage them to recognize not only the lower quality of health services in East Jerusalem, which they mentioned in the interviews, but also the structural (political, policy, economic, legal) causes of this gap which require structural solutions.

Participants de-emphasized conflict-related determinants. However, when they did refer to the Israeli-Palestinian conflict, they often mentioned the victims of Palestinian terror attacks whom they devotedly treated at the ED – a difficult and memorable experience for them. Raising the staff's awareness to additional conflict-related determinants that affect their Arab patients, determinants to which they are less directly exposed in the ED, can be an effective element in a structural competency training.

The staff members’ deeper understanding will possibly motivate them to advocate for their most disempowered patients in collaboration with them [52], fostering solidarity, social justice, and health equity [2, 21, 72]. The staff can engage in promoting varying structural-political solutions, according to their diverse perspectives, values, and beliefs. These solutions can vary from the individual patient level to the policy change level [53]. Given the need for up-to-date empirical data on the structural determinants of health that affect ED practices, ED staff can play a key role in leading research projects on these issues. The ED staff members are dedicated professionals who are committed to providing the best possible healthcare to their patients despite the ED overcrowding and staff’s overwork. Structural competency training will allow them to help their patients in additional novel ways.

## Conclusion

This article explored the tension between an ethos of medical neutrality and the recognition of difference in the treatment of Arab patients in a Jerusalem ED. Through analysis of mostly Jewish medical staff perspectives, we found that they operated with an ethos of neutrality in which they were expected to provide equal treatment regardless of patients’ national identity or ethnic and religious backgrounds. We found that participants supported the ethos of neutrality as a humanitarian approach to care, while also recognizing its limits in the context of the Israeli-Palestinian conflict. While they recognized the influence of political conflict, staff were more likely to understand the differing health status of Arab patients through depoliticized concepts of cultural and behavioral difference. We suggest that the normative demand for neutrality works to exclude discussion of the Israeli-Palestinian conflict from clinical spaces, and that the normative exclusion of politics is an important aspect of how political conflict operates as a structural determinant of health and healthcare.

## Data Availability

The qualitative data are not publicly available because they contain information that compromises research participants' privacy and consent.

## Footnotes

1 “Tipat Halav is a center that provides health and medical services in the field of health promotion and prevention for pregnant women, infants and children (from birth to age 6 years) and their families” [47].

2 We included only students employed by the hospital, not students who were trained there as part of their studies.

3 By 2017, 75% of the Palestinian families in Jerusalem were living below the poverty line, compared to 22% of Jewish families [41].
